# Chemoradiotherapy versus chemotherapy as adjuvant treatment for localized gastric cancer: a propensity score-matched analysis

**DOI:** 10.1186/s12885-018-4305-x

**Published:** 2018-04-03

**Authors:** Daniel M. Girardi, Mariana A. de Lima, Gabriel C. B. Pereira, Marcelo V. Negrão, Rossana V. M. López, Fernanda C. Capareli, Jorge Sabbaga, Paulo Marcelo G. Hoff

**Affiliations:** 1 Department of oncology, Hospital Sírio Libanês, SGAS 613, conjunto E lote 95, Asa Sul, Brasília, DF 70200-001 Brazil; 2 Instituto do Câncer do Estado de São Paulo, Faculdade de Medicina da Universidade de São Paulo, São Paulo, Brazil; 3 Oncoclinica, Macapá, Brazil

**Keywords:** Gastric cancer, Adjuvant, Propensity score, Chemoradiotherapy, Chemotherapy

## Abstract

**Background:**

Treatment of localized gastric cancer (LGC) consists of surgical resection followed by adjuvant treatment. Both chemoradiation (CRT) and chemotherapy (CT) regimens have shown benefit in survival outcomes versus observation. However, there are few data comparing these approaches.

**Methods:**

This study included consecutive patients with LGC treated at Instituto do Cancer do Estado de Sao Paulo (ICESP) from 2012 to 2015. CRT was based on the INT-0116 regimen and CT consisted of a platinum and fluoropyrimidine doublet. Treatment choice was based on physician preference. Toxicity was evaluated for every cycle. Overall survival (OS) analysis was performed by Kaplan-Meier. A propensity score-matched analysis was performed to minimize selection bias.

**Results:**

A total of 309 patients were evaluated, 227 in CRT group and 82 in CT group. The most prevalent grade 3/4 toxicities in CRT and CT groups were: nausea/vomiting (9.25 vs 4.9%), fatigue (9.3% vs 2.4%), mucositis (4.4% vs 1.2%), neutropenia (37.8% vs 20.9%), febrile neutropenia (3.9% vs 0%), anemia (4.3% vs 6.1%), thrombocytopenia (2.6% vs 4.9%), neuropathy (0 vs 2.4%) and hand-foot syndrome (0.4% vs 2.4%). Two grade 5 toxicities (febrile neutropenia and anemia) occurred in CRT group. There was no difference in the pattern of recurrence. After a median follow-up of 23.5 months (CRT) and 20.6 months (CT), there was no difference in OS between groups.

**Conclusions:**

CT and CRT present similar efficacy and tolerability as adjuvant treatment for LGC.

## Background

For the majority of patients with LGC cure is not obtained with surgery alone [1]. Recurrence is high (range: 40%-80%) [2, 3] and 5 year survival rates are around 35% and 20% for stages II and III respectively [4, 5]. The benefit of adjuvant treatment has been thoroughly studied and these therapies are now considered standard of care for patients with LGC. In the Intergroup 0116 (INT 0116) trial [6, 7], adjuvant fluoropyrimidine-based chemoradiation (CRT) significantly improved OS for LGC compared to surgery alone. Similarly, the ACTS-GC [8] and CLASSIC [9] trials have demonstrated that adjuvant chemotherapy with S-1 or capecitabine plus oxaliplatin reduced the risk of relapse and death in patients with LGC.

Clinical trials have also directly compared postoperative CRT with CT alone for patients with LGC. A meta-analysis of four randomized clinical trials with patients submitted to D2 lymphadenectomy for LGC showed that CRT significantly reduced the risk of locoregional recurrence, but without significant improvement in distant relapse and OS [10]. The ARTIST trial also compared adjuvant CT with capecitabine and cisplatin (XP) to XP followed by capecitabine-based concurrent chemoradiation. After a 7-year follow-up, the 5-year OS was similar between both groups (73 vs 75%; HR 1.130; 95% CI, 0.775 to 1.647; *P* = 0.5272) [11]. Based on these results, it remains unclear which is the optimal adjuvant treatment for LGC with no globally accepted standard of care. In this study, we have used a propensity score matched analysis to retrospectively evaluate the efficacy and toxicity of adjuvant CRT versus CT alone for treatment of LGC.

## Methods

### Patients

We retrospectively evaluated consecutive patients with LGC who received adjuvant treatment from January 2012 to November 2015 at *Instituto do Cancer do Estado de Sao Paulo* (ICESP), Sao Paulo, Brazil. The Ethics Committee of ICESP approved the study protocol. Patients were eligible for analysis if they were 18 years or older, had histological confirmation of gastric cancer, had curative gastrectomy with nodal dissection and had received adjuvant treatment. The choice of adjuvant treatment was based on physician preference. Exclusion criteria included the use of neoadjuvant treatment and metastatic disease at diagnosis.

### Adjuvant treatment

Adjuvant CRT was based on the INT 0116 trial [7, 8] with intravenous bolus 5-fluorouracil (5-FU) and leucovorin (LV) before, during, and after radiotherapy. Adjuvant CT consisted of a fluoropyrimidine-platinum doublet with options including XelOx (eight cycles of oral capecitabine 1000 mg/m2 twice daily on days 1–14 plus intravenous oxaliplatin 130 mg/m^2^ on day 1 every 3 weeks) or XP regimens (six cycles of oral capecitabine 1000 mg/m^2^ twice daily on days 1–14 plus intravenous cisplatin 80 mg/m^2^ on day 1 every 3 weeks).

### Objectives and statistical analysis

The primary endpoint of the study was to compare OS (measured from the first day of systemic treatment until death or last follow-up) for adjuvant CRT and CT alone. Secondary endpoints include treatment toxicity, loco-regional recurrence and distant recurrence.

A propensity score-matched analysis was used in order to balance the two treatment groups. The subjects were matched 1:1 according to the following covariates: tumor histology, pathological staging (I-II and III), type of lymphadenectomy and surgical margin. After this adjustment, OS was estimated using Kaplan-Meier method and log-rank test.

Data regarding treatment toxicity were recorded for each cycle of adjuvant treatment according to the National Cancer Institute - Common Toxicity Criteria (NCI-CTC, version 4.0) [12]. Frequencies were compared using chi-square and Fisher’s exact tests. Hazard ratios with 95% confidence intervals were determined with the use of a Cox proportional-hazards model. Significance level of 0.05 was considered for all the analyses and all *p* values were two-sided.

Patterns of recurrence were analyzed in both groups. Loco-regional recurrence was defined as disease recurrence in the tumor bed, anastomosis, regional lymph nodes, radiation field and/or, for those patients submitted to partial gastrectomy, the remaining stomach. Distant recurrence was defined as recurrence in the liver, peritoneum, non-regional lymph nodes or any other extra abdominal site.

## Results

### Patient characteristics

From January 2012 to November 2015, a total of 309 patients were eligible for the study, 227 in the CRT group and 82 in the CT group. Baseline characteristics are summarized in Table 1. Age, gender, performance status and tumor histology were similar between groups. Groups were not balanced regarding pathological staging (*p =* 0.021), with more stage III patients in the CT group (68.3%) than in the CRT group (52.5%). Imbalances were also observed for type of lymphadenectomy and surgical margin since D2 dissections were more frequent in CT than in CRT group (79.3% vs. 73.1%) and more microscopic tumor infiltrated margins (R1 resections) were observed in CRT than in CT group (12.2 vs. 7.9%). However, these differences were not statistically significant (*p =* 0.273 and *p =* 0.359, respectively).

**Table 1:** Patient characteristics

	Chemoradiation (*N* = 227)	Chemotherapy (*N* = 82)	*P*
Age at diagnosis – years			0.124
Median	58.71	56.51	
Range	30 - 80	34 - 75	
Sex - No. (%)			0.964
Male	135 (59.5)	49 (59.8)	
Female	92 (40.5)	33 (40.2)	
ECOG Performance status - No. (%)			0.401
0	97 (42.7)	38 (46)	
1	112 (49.3)	38 (46.3)	
2	18 (7.9)	5 (6.1)	
3	0 (0.0)	1 (1.2)	
Histology - No. (%)			0.712
Diffuse/signet ring	97 (42.7)	34 (41.5)	
Tubular/intestinal	90 (39.6)	35 (42.7)	
Not specified	40 (17.6)	13 (15.9)	
TNM Stage group - No. (%)			0.021
IB	11 (4.8)	3 (3.7)	
IIA	54 (23.8)	10 (12.2)	
IIB	43 (18.9)	13 (15.9)	
IIIA	41 (18.1)	10 (12.2)	
IIIB	42 (18.5)	22 (26.8)	
IIIC	36 (15.9)	24 (29.3)	
Lymph node dissection - No. (%)			0.273
D1	61 (26.9)	17 (20.7)	
D2	166 (73.1)	65 (79.3)	
Surgical margin - No. (%)			0.359
R0	207 (91.2)	72 (87.8)	
R1	18 (7.9)	10 (12.2)	
R2	2 (0.9)	0 (0.0)	

### Adjuvant treatment

All 227 patients in the CRT group were treated with the INT-0116 trial^7^ regimen. In the CT group, 78 patients (95.1%) were treated with capecitabine and oxaliplatin and 4 patients (4.9%) received capecitabine and cisplatin.

### Efficacy

**Fig. 1:**
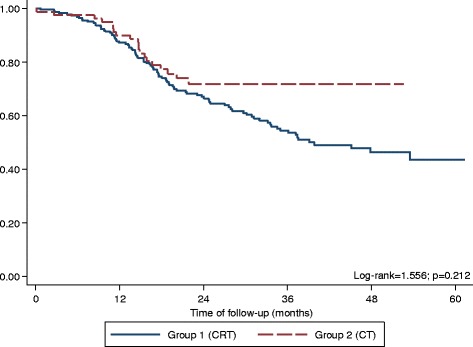
Kaplan-Meier curves for overall survival in the study population (*N* = 309)

**Fig. 2:**
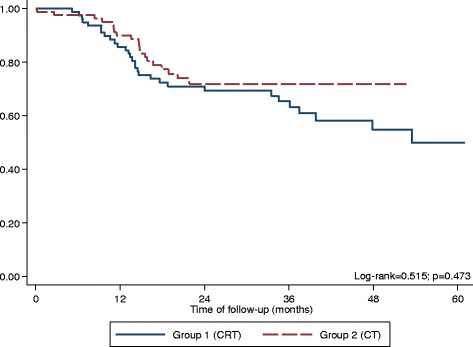
Kaplan-Meier curves for overall survival according to *Propensity score* analysis (*N* = 162)

After a median follow-up time of 23.5 months (range 0.7-61.3 months) in the CRT group and 20.6 months (range 0.2-52.6 months) in the CT group, the two-year OS was similar between groups (67.1% vs 71.9% for CRT and CT groups respectively). Median OS was 39.8 months for the CRT group and not reached in the CT group (HR 0.73; 95% CI, 0.45 to 1.19; *p* = 0.212) (Fig. 1). A 1:1 propensity score-matched analysis using histologic type, pathological staging, type of lymphadenectomy and surgical margin was performed, resulting in a total of 162 patients, 80 in the CRT group and 82 in the CT group. There was no significant difference between the two treatment groups in median OS (53.5 months vs not reached for CRT and CT groups respectively; HR 0.80; 95% CI, 0.44 to 1.45; *p* = 0.47) (Fig. 2).

### Toxicity

The toxicity of each treatment is summarized in Table 2. The most common events in the CRT group were gastro-intestinal, such as nausea/vomiting, diarrhea and mucositis, and hematological, such as neutropenia, anemia and thrombocytopenia. Similarly, gastro-intestinal and hematological adverse events were also the most prevalent in the CT group, followed by neuropathy and hand-foot syndrome. The most common grade 3-4 adverse events in the CRT group were neutropenia (37.8%), diarrhea (10.1%), fatigue (9.3%) and nausea/vomiting (9.2%), while in the CT group neutropenia (20.8%), diarrhea (9.7%), anemia (6.1%) and thrombocytopenia (4.9%) were the most common events. Two patients died in the CRT group as a consequence of treatment related adverse events. One patient died because of complications related to febrile neutropenia and another one due to severe anemia. There were no treatment related deaths in the CT group.

**Table 2:** Adverse events

	Chemoradiation (*N* = 227)					Chemotherapy (*N* = 82)				
Toxicity	Grade 1	Grade 2	Grade 3	Grade 4	Grade 5	Grade 1	Grade 2	Grade 3	Grade 4	Grade 5
	No. (%)	No. (%)	No. (%)	No. (%)	No. (%)	No. (%)	No. (%)	No. (%)	No. (%)	No. (%)
Alopecia	7 (3,1)	2 (0,9)	–	–	–	–	–	–	–	–
Nausea/vomiting	92 (40.5)	47 (20.2)	20 (8.8)	1 (0.4)	–	34 (41.5)	25 (30.5)	4 (4.9)	–	–
Diarrhea	91 (40.1)	40 (17.6)	20 (8.8)	3 (1.3)	–	35 (42.7)	16 (19.5)	6 (7.3)	2 (2.4)	–
Anorexia	46 (20.3)	38 (16.7)	18 (7.9)	–	–	10 (12.2)	7 (8.5)	1 (1.2)	–	–
Asthenia	47 (20.7)	23 (10.1)	21 (9.3)	–	–	16 (19.5)	22 (26.8)	2 (2.4)	–	–
Dysphagia	7 (3.1)	2 (0.9)	3 (1.3)	–	–	2 (2.4)	2 (2.4)	–	–	–
Neuropathy	–	–	–	–	–	38 (46.3)	18 (22)	2 (2.4)	–	–
Mucositis	37 (16.3)	22 (9.7)	9 (4.0)	1 (0.4)	–	17 (20.7)	2 (2.4)	–	1 (1.2)	–
Neutropenia	16 (7.0)	34 (15.0)	48 (21.1)	38 (16.7)	–	4 (4.9)	20 (24.4)	14 (17.1)	3 (3.7)	–
Febrile neutropenia	–	–	6 (2.6)	2 (0.9)	1 (0.4)	–	–	–	–	–
Anemia	58 (25.6)	29 (12.8)	8 (3.5)	1 (0.4)	1 (0.4)	48 (58.5)	16 (19.5)	5 (6.1)	–	–
Thrombocytopenia	54 (23.8)	14 (6.2)	5 (2.2)	1 (0.4)	–	29 (35.4)	6 (7.3)	4 (4.9)	–	–
Renal toxicity	6 (2.6)	3 (1.3)	2 (0.9)	–	–	3 (3.7)	3 (3.7)	–	1 (1.2)	–
Hand-foot syndrome	7 (3.1)	–	1 (0.4)	–	–	30 (36.6)	5 (6.1)	2 (2.4)	–	–

Dose reductions were more common in the CT than in CRT group (52.4% vs. 11%). Treatment delays for more than 7 days were similar between groups (29% vs 32.9% for CRT and CT group respectively), as well as treatment discontinuation rates (35.7% vs 35.4%). The most common cause of treatment discontinuation in the CRT group was toxicity (48.2%) followed by disease progression (19.7%) and loss of follow up (18.5%). For the CT group, treatment discontinuation was due to toxicity (41.4%), followed by disease progression (20.7%) and patient preference (17.2%).

### Pattern of recurrence

There was no significant difference in the pattern of recurrence between groups (*p =* 0.662). Systemic recurrence (76.9% vs 69.7%, for CRT and CT respectively) was more common than locoregional recurrence (17.6% vs 21.2%, for CRT and CT respectively) in both groups (Table 3).

**Table 3:** Recurrence patterns

	Chemoradiation (*N* = 227)	Chemotherapy (*N* = 82)	*P*
			0.662
Loco-regional - No. (%)	16 (17.6)	7 (21.2)	
Systemic - No. (%)	70 (76.9)	23 (69.7)	
Loco-regional and systemic - No. (%)	5 (5.5)	3 (9.1)	

## Discussion

Although it is widely accepted that complete resection of the tumor with adequate margins is the appropriate surgical treatment for LGC, controversy remains regarding extension of nodal dissection and best adjuvant treatment. In Eastern countries, gastrectomy with D2 lymph node dissection has been the standard surgical procedure for patients with LGC for several decades. Based on significantly lower loco-regional recurrence rates and gastric cancer-related deaths with D2 dissection [13], this type of surgery is now recommended in the United States and Europe as well [14, 15].

Our study aimed to compare two distinct adjuvant strategies for the treatment of LGC: CRT and CT. Most of our patients had D2 lymph node dissection, which is the standard at our institution. Most patients with D1 lymph node dissection had surgery at outside institutions and were referred to our service for adjuvant treatment. Our results show no difference in OS between the two groups. Loco-regional recurrence rates were also similar between groups (17.6% vs 21.2%) and, as expected, systemic relapse was the most common site of recurrence for the majority of patients (76.9% vs 69.7% for CRT and CT groups respectively; *p* = 0.662).

In the INT-0116 trial, only 10% of patients underwent D2 dissection [8]. Therefore, it is possible that radiation in this trial might only have compensated for sub-optimal lymph node dissection, decreasing loco-regional recurrence, and leaving the role of adjuvant radiation for D2-dissected patients uncertain. Two trials evaluated the role of adjuvant CT in D2-dissected patients [9, 10]. The pivotal Japanese ACTS-GC study evaluated post-operative S-1, an oral fluoropyrimidine with a 5-FU prodrug and two modulators of 5-FU metabolism, in patients who underwent D2 gastrectomy, and reported a 5-year OS benefit in the S-1 arm (71.7% vs 61.1%; HR 0.67; 95% CI, 0.540 to 0.828) [9]. In the CLASSIC trial, patients with stage II-IIIB gastric cancer also submitted to D2 gastrectomy were randomly assigned to either CT or observation. The 5-year disease-free survival was 68% for the experimental arm versus 53% in the surgery alone arm (HR 0.58, 95% CI 0.47–0.72; *p* < 0.0001) [16]. These trials show that D2 gastrectomy followed by adjuvant CT led to a survival benefit when compared to surgery alone, but they did not include a head-to-head comparison of CRT and CT.

In a retrospective study conducted in D2-dissected Chinese patients, adjuvant CRT (INT-0116 regimen) and CT alone (fluoropyrimidine alone or in combination with oxaliplatin) had similar efficacy with a median OS of 51.0 vs 48.6 months respectively (*p* = 0.251) [17]. The ARTIST trial [12] prospectively compared adjuvant CRT and CT alone for D2-dissected LGC and also failed to show a survival difference between these two strategies. 5-year OS was 75% vs 73% for the CRT and CT arms respectively (*P =* 0.484). However, in a subgroup analysis of patients with pathologic lymph node involvement, the 3-year disease-free survival was prolonged in the CRT arm compared with the chemotherapy arm (77.5% vs. 72.3%; *P =* 0.0365). A meta-analysis with a total of 960 Asian patients from four randomized clinical trials of D2-resected LGC also did not find an OS difference between CRT and CT alone (*P* = 0.34), despite adjuvant CRT significantly improving loco-regional recurrence-free survival (HR 0.50, *p* = 0.0005) and disease-free survival (HR 0.73, *P* = 0.002) [11]. This shows that systemic disease was probably the main cause of relapse in these patients making adequate control of micrometastatic disease critical in these patients. The CRITICS trial evaluated patients undergoing platinum-fluoropyrimidine CT followed by D1+ gastrectomy and subsequently randomized to CRT or CT. Both groups had similar median OS and disease-free survival, with 5-year OS of 40.9% vs 41.3% for CRT and CT respectively (*p* = 0.99) [18]. Opposite findings were shown in a recent large retrospective trial using the National Cancer Database (NCDB). This study evaluated 3656 patients with LGC treated with perioperative CT without radiation versus adjuvant CRT. The results demonstrated improved median OS with adjuvant CRT (51 months for CRT vs 42 months for perioperative CT; *p* = 0.013). However, there were several imbalances in the study population and, after propensity score-matched analysis, the benefit remained significant, but much less remarkable (5-year OS 45% vs 42%; HR 0.886; 95% CI 0.793-0.990, *p* = 0.033) [19].

In our study, the most common grade 3-4 toxicities in the CRT group were neutropenia (37.8%) and gastrointestinal (approximately 10%), similar to data reported in the INT-0116 trial [7]. In the CT group, the most common grade 3-4 toxicities were neutropenia (20.8%), diarrhea (9.7%), anemia (6.1%) and thrombocytopenia (4.9%), which is similar to the toxicity profile of the CLASSIC trial [10]. Dose reductions and dose delays were more common in patients treated with CT than with CRT, but treatment discontinuation rates were similar between groups. In the CLASSIC trial, dose reductions due to adverse events were seen in 90% of patients and treatment discontinuation rate was 10% [10]. On the other hand, in the INT-0116 trial only 65% of patients completed CRT as planned [7]. Two grade 5 toxicities occurred in the CRT arm, which is also similar to previous studies [7].

Our study has limitations. It is a retrospective study which leads to higher risk of selection bias. Indeed the two groups had different TNM pathological stage distributions, with more stage III patients in the CT group than in CRT group (68.3% vs. 52.5%; *p =* 0.021). This reflects the current practice at our institution of prioritizing more aggressive systemic therapy in case of extensive nodal disease and bulky primary tumors, which carry a higher risk for systemic recurrence. Moreover, the CT alone group had a slightly higher proportion of patients with D2-resected tumors (79.3% versus 73.1% for CT and CRT respectively), probably due to the fact that this population did not derive benefit from radiation in previous randomized trials [9, 10]. However, these possible sources of selection bias were contemplated through propensity score-matched analysis which included histology, pathological staging, type of lymphadenectomy and surgical margin as covariates. The results confirmed the data from the whole population of study and showed no difference in OS between CRT and CT groups (Fig. 2).

Despite recent advances, gastric cancer is still associated with a high risk of disease relapse and mortality and research is ongoing to determine optimal treatment strategies. In a follow-up of the ARTIST trial, ARTIST-II is comparing CRT and CT in in the high-risk lymph node-positive population (NCT01761461). Final results of the CRITICS trial (NCT00407186) are also awaited and a Chinese phase III trial is recruiting participants to compare adjuvant CT with S1 and oxaliplatin versus adjuvant CRT (NCT02648841). The TOPGEAR trial (NCT01924819) is currently evaluating the role of neoadjuvant CRT to increase treatment efficacy and compliance and reduce toxicity. Lastly, final results of FLOT4-AIO Trial (NCT01216644) are eagerly awaited due to a survival and pathological complete response benefit of perioperative docetaxel, oxaliplatin and 5-FU (FLOT regimen) over standard platinum, fluoropyrimidine and anthracycline regimens [20, 21], which could change the standard of care in this setting.

Another field of active research is tumor biology. Data from the FLOT4 trial suggest that perioperative CT has limited activity for diffuse histology tumors (0% pathological complete response rate) [21]. In a retrospective analysis of the MAGIC trial, microsatellite instability was associated with lack of benefit, and maybe even harm, from perioperative systemic treatment [22]. PD-L1 expression has been used in the recent development of immunotherapy agents with promising results in the metastatic setting [23]. Recent data from Le et al. suggests that for tumors with microssatellite instability [24, 25], PD-1 checkpoint inhibition is a game changer, with high response rates and durable responses, which led to FDA approval in this setting. Lastly, initiatives such as The Cancer Genome Atlas (TCGA) [26] have helped to advance our knowledge of tumor biology and it is expected that tumor genomic profiling will play a more robust role in clinical decisions and development of clinical trials in the near future. As an example, recent data from a retrospective study suggests that the molecular classification of gastric cancers proposed by TCGA could help predict outcomes and even benefit from adjuvant chemotherapy [27].

## Conclusion

Our study shows that adjuvant CRT and CT alone present similar efficacy and manageable toxicity profile even though dose reductions and dose delays were more common in patients treated with CT alone. Therefore, in institutions where radiation therapy is a scarce resource, adjuvant CT alone is a reasonable treatment option. Research is ongoing to determine more effective treatment strategies and to improve patient selection.

## Abbreviations

CRT: Chemoradiation
CT: Chemotherapy
ICESP: Instituto do Cancer do Estado de Sao Paulo
LGC: Localized gastric cancer
NCDB: National cancer database
OS: Overall survival
TCGA: The cancer genome atlas
